# Supplementary material for: Phylogenomic subsampling and upsampling for efficient evolutionary analyses of big data
Source: Mol Biol Evol. 2026 Aug 21;43(9):msag218. doi: 10.1093/molbev/msag218 (PMC13551623; doi:10.1093/molbev/msag218)
Supplement: Supplementary file 1 [file msag218_supplementary_data.zip › Appendix-FINAL.pdf]

Appendix

Median versus Mean for Binary Scoring in Bootstrap Analysis

The little bootstrap framework requires aggregation of subsample-wise estimates. While Kleiner et al. (2014) focused primarily on mean bagging, Sharma and Kumar (2021) found that median bagging yields estimates closer to conventional bootstrap support. To illustrate why median bagging is appropriate, we considered an indicator-bootstrap framework analogous to a phylogenetic bootstrap analysis. In this setting, each bootstrap replicate receives a binary score: 1 if it supports a hypothesis and 0 otherwise. The bootstrap estimate is then the proportion of replicates that have a score of 1. This parallels phylogenetic bootstrapping, where a clade receives a score of 1 when present in a replicate tree and 0 when absent.

We generated data from a normal distribution and compared p-values obtained using the standard bootstrap with those obtained using the bag-of-little-bootstraps framework. For the little bootstrap analyses, subsamples of size (N^g) were analyzed using both mean and median bagging. Median bagging consistently produced estimates that more closely matched those obtained from the standard bootstrap (**Fig. A1**). Mean bagging was more sensitive to a small number of subsamples that strongly supported or rejected the hypothesis. These extreme subsamples tend to pull the average toward 0.5, the expected value under random support. Consequently, mean bagging tends to overestimate small *P*-values and underestimate large p-values. A similar pattern was observed for phylogenetic bootstrap support values (Sharma and Kumar 2021), where median bagging yielded *BS* values that more closely approximated conventional full-alignment bootstrap support (*FBS*).

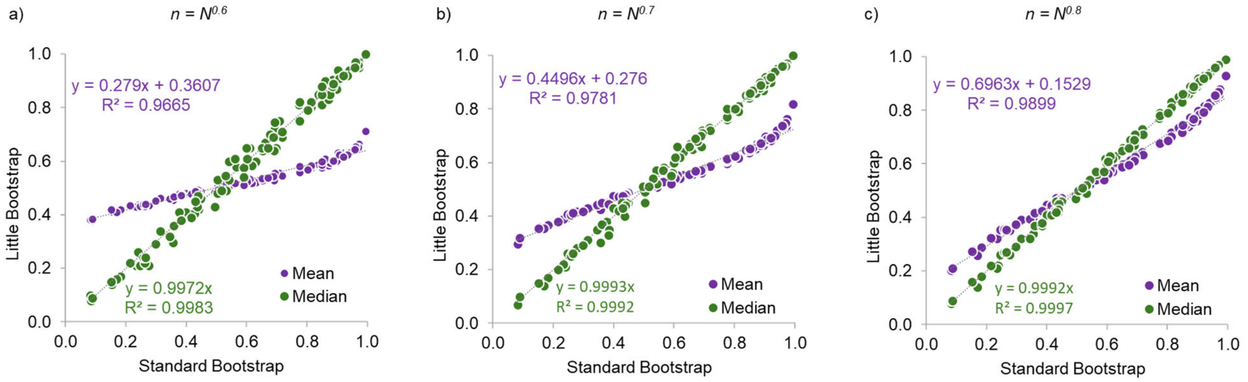

Figure A1. Comparison of p-values estimated by the standard and little bootstrap with mean (blue) and median bagging (orange).

Data were simulated from a normal distribution X ~ *Normal*(μ = −5, σ = 1). We generated 100 random samples of size N = 25,000 and tested H_0: μ = −5.01 against the one-sided alternative H_1: μ < −5.01, a threshold chosen to produce a broad range of p-values. For each sample, p-values were estimated using the standard bootstrap and the bag-of-little bootstraps. In the little bootstrap, subsamples of size $n = N^g$ were drawn, with g = 0.6, 0.7, or 0.8 in panels **a**-**c**, respectively. For each value of g, 1000 subsamples were analyzed, and 100 upsampled replicates were generated from each subsample. Subsample-wise p-values were then aggregated using either the mean or the median.
