# Supplementary material for: Phylogenomic subsampling and upsampling for efficient evolutionary analyses of big data
Source: Mol Biol Evol. 2026 Aug 21;43(9):msag218. doi: 10.1093/molbev/msag218 (PMC13551623; doi:10.1093/molbev/msag218)
Supplement: Supplementary file 1 [file msag218_supplementary_data.zip › Supplementary Table S1-FINAL.pdf]

Supplementary Table S1. Information for datasets in Figure 1.

Data	Base type	Taxa	Total Sites	Unique Site patterns (*U*)	Data Size (*D*)	Median pairwise difference (%) (Minimum - Maximum)	DOI
Insects A	DNA	174	3,011,544	2,045,783	355,966,242	13.9 (0.3 - 30.1)	https://doi.org/10.1016/j.cub.2017.01.027
Butterflies	DNA	61	5,267,461	3,762,723	229,526,103	21.9 (1.1 - 28.3)	https://doi.org/10.1093/sysbio/syz030
Vertebrate	AA	58	1,806,035	1,547,914	8,977,9012	22.4 (2.1 - 30.3)	https://doi.org/10.1093/sysbio/syv059
Plants A	AA	360	19,449	190,352	68,526,720	3.8 (0.0 - 18.9)	https://doi.org/10.1186/1471-2148-14-23
Insects B	DNA	48	2,938,039	1,396,402	67,027,296	15.1 (3.2 - 21.1)	https://doi.org/10.1016/j.ympev.2017.12.005
Fungi	AA	86	609,772	562,771	48,398,306	51.2 (0.4 - 59.7)	https://doi.org/10.1038/s41559-017-0126
Birds	DNA	200	394,684	226,490	45,298,000	7.4 (0.5 - 16.6)	https://doi.org/10.1038/nature15697
Mammal A	DNA	37	1,391,742	775,579	28,696,423	12.8 (0.5 - 26.5)	https://doi.org/10.1073/pnas.1211733109
Yeast	AA	23	634,530	390,960	8,992,080	41.9 (5.7 - 52.6)	https://doi.org/10.1038/nature12130
Rodents	DNA	37	1,207,638	96,293	3,562,841	2.4 (0.2 - 5.8)	https://doi.org/10.1093/sysbio/syz044
Mammal B	DNA	274	7,370	4,303	1,179,022	15.2 (0.3 - 22.2)	https://doi.org/10.1098/rspb.2012.0683
Plants B	DNA	16	4,246,454	17,789	284,624	4.8 (0.4 - 15.4)	https://doi.org/10.1016/j.ympev.2018.08.011
Lassa Virus	DNA	179	3,186	1,475	264,025	21.1 (0.0 - 24.7)	https://doi.org/10.1016/j.cell.2015.07.020

Note. Dataset size (*D*) was calculated as the product of the number of taxa and the number of unique site patterns (*U*), because both factors are associated with the computational time and memory required for maximum likelihood analysis. The median, minimum, and maximum pairwise sequence difference per site represents the level of sequence diversity within each alignment. DOI provides the digital object identifiers of the studies in which these datasets were originally published and analyzed.
